# Comparison of Enzymatic and Non-Enzymatic Means of Dissociating Adherent Monolayers of Mesenchymal Stem Cells

**DOI:** 10.1007/s12575-009-9001-4

**Published:** 2009-03-10

**Authors:** Boon C Heng, Catherine M Cowan, Shubhayu Basu

**Affiliations:** 1 New Business Ventures, Abbott Vascular, 3200 Lakeside Dr., Santa Clara, CA 95054, USA; 2 Abbott Vascular, 3200 Lakeside Dr., Santa Clara, CA 95054, USA

**Keywords:** Dissociation, Enzyme, Mesenchymal, Stem cells, Trypsin

## Abstract

The dissociation of adherent mesenchymal stem cell (MSC) monolayers with trypsin and enzyme-free dissociation buffer was compared. A significantly lower proportion of viable cells were obtained with enzyme-free dissociation buffers compared to trypsin. Subsequently, the dissociated cells were re-seeded on new cell culture dishes and were subjected to the MTT assay 24 h later. The proportion of viable cells that reattached was significantly lower for cells obtained by dissociation with enzyme-free dissociation buffer compared to trypsin. Frozen–thawed MSC displayed a similar trend, yielding consistently higher cell viability and reattachment rates when dissociated with trypsin compared to enzyme-free dissociation buffer. It was also demonstrated that exposure of trypsin-dissociated MSC to enzyme-free dissociation buffer for 1 h had no significant detrimental effect on cell viability.

## 1. Introduction

Bone marrow-derived mesenchymal stem cells (MSC) have demonstrated tremendous potential in the emerging field of regenerative medicine [1-4]. Nevertheless, a major challenge faced in the clinical application of MSC is the need for adequate cell numbers for achieving optimal efficacy in transplantation therapy. Hence, MSC need extensive ex vivo proliferation within prolonged durations of in vitro culture [5,6]. To avoid pathogenic transmission, it is imperative to minimize animal and human-derived products within MSC culture [7,8]. One key animal-derived product is the digestive enzyme trypsin, which is routinely used to dissociate adherent MSC monolayers into single-cell suspensions during serial passages. Alternative enzyme-free methods for dissociating adherent cell monolayers have been developed, such as the various commercially available enzyme-free dissociation buffers [9,10] that work on the principle of chelating free calcium and magnesium ions in solution; as well as recombinant trypsin-like proteolytic enzymes produced from bacterial fermentation (i.e., TrypLE^®^ Express commercially available from Gibco, Gaithersburg, MD, USA). However, it must be noted that enzymatic cell dissociation inevitably results in some degradation of surface proteins and glycoproteins. Hence, enzyme-free cell dissociation is instead sometimes preferred to preserve the structural integrity of membrane surface proteins for ligand binding flow cytometry and immunohistochemistry [11,12].

This study compares the dissociation of adherent MSC monolayers with either enzyme-free dissociation buffer or trypsin, the most commonly used enzyme for the dissociation of in vitro cultured cells. The viability of the newly dissociated cells was assessed by a simple trypan blue exclusion assay through the use of an automated cell counter. Subsequently, the dissociated MSC was re-plated on new cell culture dishes and subjected to the MTT assay 24 h later, to assess the re-attachment of viable cells. Additionally, similar assays were conducted on frozen–thawed MSC that were dissociated either with trypsin or enzyme-free dissociation buffer.

## 2. Materials and Methods

### 2.1. Cell Viability Assessment

Bone marrow-derived human MSC (Cat no: PT-2501, batch no: 6F4382, cryopreserved at the second passage) were purchased from Lonza (Walkersville, MD, USA). Cryopreserved MSC were thawed and cultured up to five passages upon purchase from Lonza (Walkersville, MD, USA), prior to being utilized for this study. Confluent monolayers of MSC cultured within 12-well cell culture dishes (≈1.0–1.5 × 10^5^ cells per well, surface area ≈ 4.8 cm^2^) were then dissociated with either 0.05% (*w*/*v*) Trypsin–EDTA (0.53 mM EDTA × 4 Na, Cat no. 25300-054; Gibco BRL, Gaithersburg, MD, USA) or enzyme-free phosphate-buffered saline (PBS)-based cell dissociation buffer (Cat no. 13151-014; Gibco BRL, Gaithersburg, MD, USA). According to the manufacturer's specification, the enzyme-free cell dissociation buffer is a membrane-filtered, isotonic, and enzyme-free aqueous formulation of salts, chelating agents, and cell-conditioning agents constituted in Ca^2+^- and Mg^2+^-free PBS. Prior to cell dissociation, both trypsin and the enzyme-free cell dissociation buffer were pre-warmed to 37°C within a water bath. The confluent monolayers of MSC cultured within 12-well cell culture dishes were then washed two times with Ca^2+^-free PBS, prior to the addition of 1 ml trypsin solution or enzyme-free cell-dissociation buffer within each well. These were then placed within the cell culture incubator and subjected to gentle pipetting every 2–3 min. On average, dissociation of confluent MSC monolayers with trypsin takes approximately 5–6 min with gentle pipetting; while with enzyme-free cell dissociation buffer, the corresponding duration is approximately 15 to 16 min. All pipeting was carried out with an automated pipet pump (VWR, Brisbane, CA, USA), and care was taken to ensure that the pipeting force was similar for both experimental groups, by utilizing the same settings and similar volume pipettes. The dissociated cell suspension from each well of the 12-well dish [(1.17 ± 0.06) × 10^5^ cells in 1 ml] were then placed in 1.5 ml microcentrifuge tubes and subjected to centrifugation at 500×*g* for 5 min. The supernatant was discarded, and the cell pellet was reconstituted in 0.5 ml PBS (with Ca^2+^), placed within accessory sample vials (Cat no. 383721, Beckman-Coulter, Fullerton, CA, USA) and analyzed for cell viability with the trypan blue exclusion assay, by utilizing an automated cell counter (Vi-Cell^®^ XR analyzer, Cat no. 383556; Beckman Coulter, Fullerton, CA, USA) and Vi-CELL^®^ XR Quad Pak Reagent Kit (Cat no. 383198; Beckman-Coulter, Fullerton, CA, USA). The automated cell counter mixes the cell suspension with an equal volume of 0.4% (*w*/*v*) trypan blue solution (500 μl), and automatically accounts for the fold dilution with trypan blue solution. For each experimental group, there were three replicate readings.

### 2.2. MTT Assay on Reattached Cells

MSC were seeded in 12-well cell culture dishes with 5.0 × 10^4^ cells per well (≈4.8 cm^2^). After 5 to 6 days of culture, confluent MSC monolayers were attained (1.17 × 10^5^ cells per well), and these were then dissociated with either trypsin or enzyme-free dissociation buffer. Some wells containing intact confluent MSC monolayers were retained as the control and were not dissociated with either trypsin or enzyme-free cell dissociation buffer. The dissociated cell suspension from each well were collected and placed within microcentrifuge tubes and subjected to centrifugation at 500×*g* for 5 min. The supernatant was then discarded, and the cell pellet was reconstituted in 1.0 ml of cell culture media (MSCGM^®^ bullet kit, Cat no. PT-3001; Lonza, Walkersville, MD, USA) prior to being re-seeded onto fresh 12-well cell culture dishes. After 24 h of culture, the unattached cells were washed off with PBS, and the reattached cells were then subjected to the MTT (3-(4,5-dimethylthiazol-2-yl)-2,5-diphenyltetrazolium bromide) assay [13]. Briefly, this involved placing 1.0 ml of 1 mg/ml MTT constituted in culture media within each well, following by incubation for 3 h at 37°C in the dark. After incubation, the MTT solution was removed, and the stained cells were washed two times in PBS followed by air-drying. The MTT-formazan products were extracted in the dark at room temperature with 0.25 ml of DMSO in each well. One hundred microliters aliquots of the supernatant in each well were then transferred into a 96-well flat-bottomed cell culture plates, and the absorbance was measured spectrophotometrically at 570 nm using a SpectraMax M5 modular microplate reader (Molecular Devices Corporation, Sunnyvale, CA, USA). From the absorbance values, the percentage of reattached viable cells (after dissociation with trypsin and cell-free dissociation buffer) can then be computed by dividing the MTT absorbance values obtained after dissociation with the absorbance reading for the nondissociated control (after correction for 100 μl DMSO blanks).

### 2.3. Effects of Cryopreservation on Cell Viability and Reattachment

Trypan blue exclusion and MTT assays were repeated on frozen–thawed MSC that had been dissociated either with trypsin or enzyme-free dissociation buffer. The cryopreservation solution was composed of DMEM supplemented with 10% (*v*/*v*) fetal calf serum (FCS) and 10% (*v*/*v*) DMSO. The dissociated MSC were suspended in 1 ml of cryopreservation solution within cryovials and were subjected to slow cooling within a -80°C refrigerator, through the use of isopropanol freezing containers (Nalgene, Rochester, NY, USA). After 2 h, the frozen cell-suspension within cryovials were immersed and stored in the vapor phase of liquid nitrogen for 1 h, prior to quick thawing within a water bath at 37°C.

### 2.4. Effects of Prolonged Exposure to Enzyme-free Dissociation Buffer

MSC dissociated with trypsin were exposed to enzyme-free dissociation buffer at 37°C (2.0 × 10^5^ cells per ml) for 1 h within 15 ml polypropylene tubes (Becton-Dickinson) that do not allow cell attachment. Viability of MSC in free suspension, before and after exposure to the enzyme-free dissociation buffer was assessed by the trypan blue exclusion assay, utilizing an automated cell counter.

### 2.5. Statistical Analysis of Data

There were three replicates for each experimental group, and the results from each data set were expressed as mean ± standard derivations. Differences between data sets were assessed by the paired Student's *t* test, with a value of *p* < 0.05 being considered significantly different.

## 3. Results

### 3.1. Cell Viability Assessment

**Figure 1:**
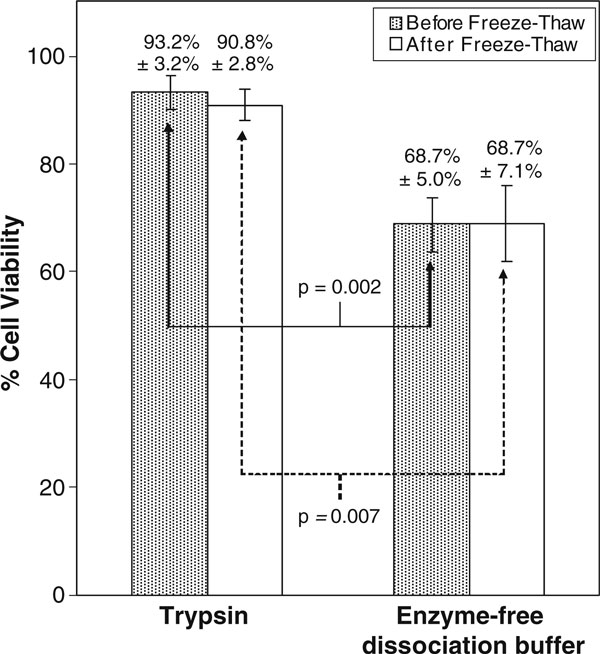
**Proportion of viable MSC (as determined by trypan blue exclusion assay) upon dissociation with trypsin and enzyme-free dissociation buffer, before and after freeze–thawing in 10% (*v*/*v*) DMSO**.

The proportion of viable MSC was significantly higher (*p* = 0.002) upon dissociation with trypsin (93.2% ± 3.2%) compared to enzyme-free dissociation buffer (68.7% ± 5.0%), as seen in Figure 1. The same trend was observed after the dissociated MSC were subjected to freeze–thawing (90.8% ± 2.8% versus 68.7% ± 7.1%, respectively, *p* = 0.007). Immediately after freeze–thawing, there was no significant reduction in the viability of MSC dissociated either with trypsin (93.2% ± 3.2% versus 90.8% ± 2.8%, p > 0.05) or enzyme-free dissociation buffer (68.7% ± 5.0% versus 68.7% ± 7.1%, *p* > 0.05).

### 3.2. MTT Assay on Reattached Cells

**Figure 2:**
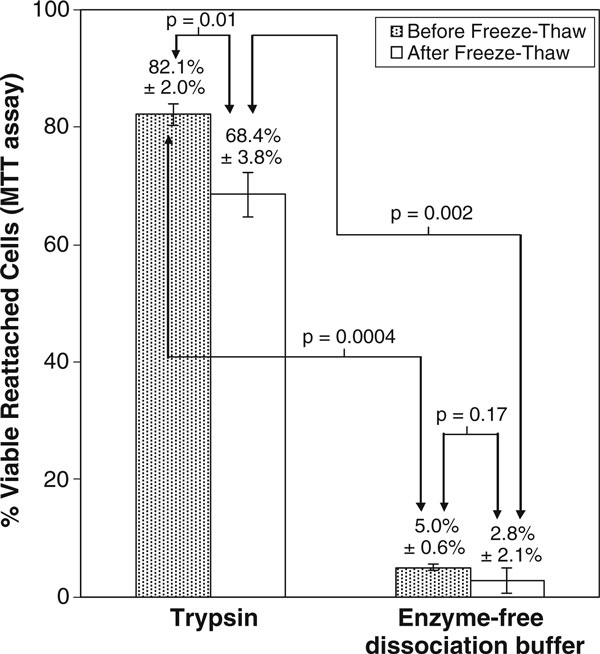
**Proportion of viable reattached MSC upon dissociation with trypsin and enzyme-free dissociation buffer, with and without freeze–thawing in 10% (*v*/*v*) DMSO**. This was assessed by MTT assay, 24 h after re-plating the dissociated cells.

**Figure 3:**
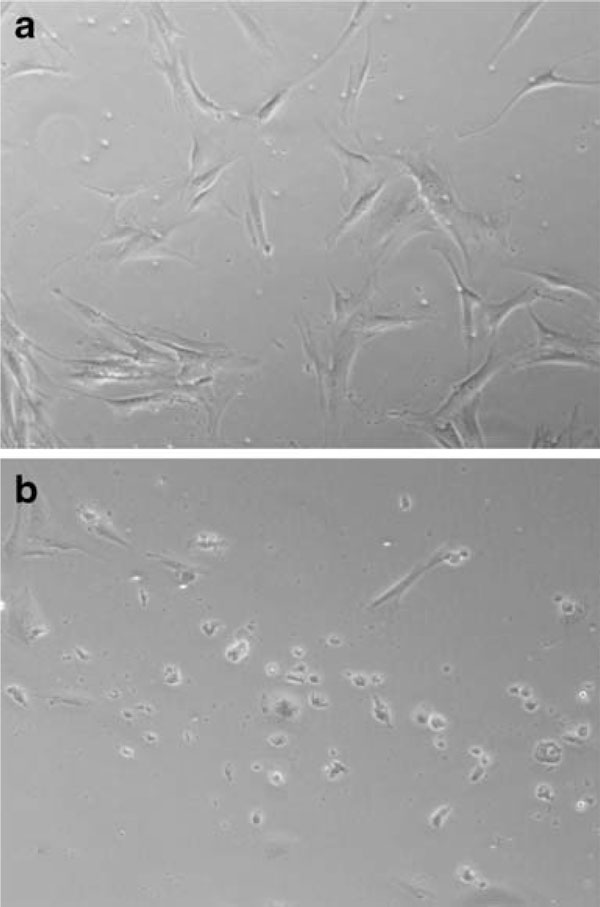
**Re-plated MSC after 24 h of culture upon dissociation with a Trypsin and b enzyme-free dissociation buffer**.

As seen in Figure 2, the proportion of viable MSC that re-attached was significantly higher (*p* = 0.0004) upon dissociation with trypsin (82.1% ± 2.0%) compared to enzyme-free dissociation buffer (5.0% ± 0.2%). The same trend was observed after the dissociated MSC were subjected to freeze–thawing (68.4% ± 3.8% versus 2.8% ± 0.4%, respectively, *p* = 0.002). As seen in Figure 3, there was a higher proportion of attached cells 24 h after reseeding MSC dissociated by trypsin than with enzyme-free buffer. Virtually, all the non-attached cells were confirmed to be nonviable by manual trypan-blue staining (data not shown). Freeze–thawing significantly reduced the proportion of viable reattached MSC upon dissociation with trypsin (82.1% ± 2.0% versus 68.4% ± 3.8%, *p* = 0.01), but not with enzyme-free dissociation buffer (5.0 ± 0.6% versus 2.8% ± 2.1%, *p* = 0.17).

### 3.3. Effects of Prolonged Exposure to Enzyme-free Dissociation Buffer

**Figure 4:**
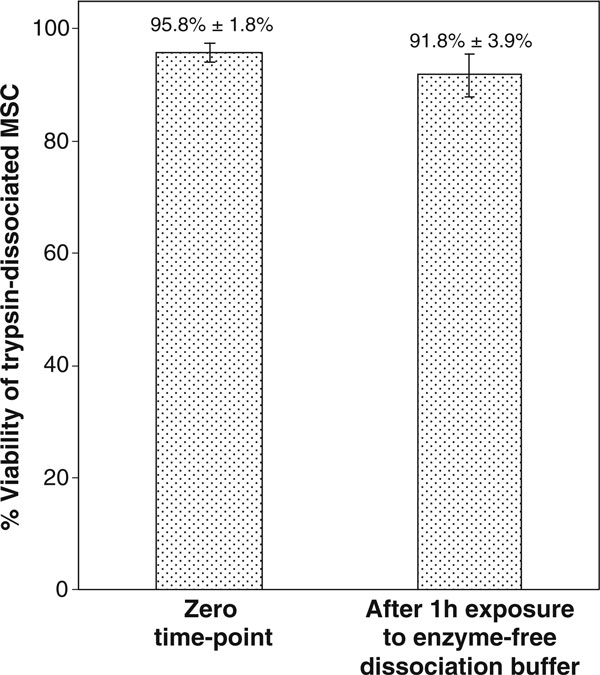
**Proportion of viable MSC (in free suspension) after dissociation with trypsin and incubation for 1 h in enzyme-free dissociation buffer**.

As seen in Figure 4, there was no significant change in cell viability after exposure of trypsin dissociated MSC to enzyme-free dissociation buffer for 1 h, (95.8% ± 1.8% versus 91.8% ± 3.9%, *p* > 0.05)

## 4. Discussion

To avoid the proteolytic effect of trypsin, commercially available enzyme-free cell dissociation buffers are sometimes utilized to preserve the structural integrity of membrane surface proteins for ligand binding flow cytometry and immunohistochemistry [11,12]. Nevertheless, enzyme-free dissociation buffers are not commonly used for the routine serial passage of various primary cell cultures and highly adherent cell lines; despite their potential to be "more gentle" on cells and avoid the potentially damaging proteolytic effects of trypsin on cell surface proteins. To our knowledge, there has not yet been any study to date that systematically investigates and compares the dissociation of confluent MSC monolayers with trypsin and enzyme-free dissociation buffer.

The results of our study showed that the immediate cell viability (trypan blue) was significantly higher upon dissociation with trypsin, compared to enzyme-free dissociation buffer. This was not because exposure of MSC to the enzyme-free dissociation buffer itself had any detrimental effect on cell viability, as demonstrated by the control experiment (Figure 4). Neither is it because of the sudden removal of magnesium and calcium ions through EDTA chelation; because the MSC monolayers were washed with calcium and magnesium free PBS prior to dissociation, and the trypsin solution itself contains EDTA (0.53 mM EDTA × 4 Na) in Ca^+2^- and Mg^+2^-free Hanks balanced salt solution. The lower cell viability with enzyme-free dissociation buffer compared to trypsin is thus manifested immediately upon dissociation and is caused neither by deprivation of free calcium ions, nor by prolonged exposure to the enzyme-free buffer per se. Future work is needed to uncover the underlying mechanisms behind these observations. In any case, it can be inferred that dissociation with the enzyme-buffer is more detrimental to MSC viability compared to trypsin. TrypLE^®^ Express was not examined in this study because of the lack of information on the proprietary protease used in it. It is claimed by the manufacturer to strongly resemble trypsin and was therefore expected to produce results comparable to that obtained by Trypsin.

Additionally, the MTT assay showed a stark decrease in the proportion of viable reattached MSC upon dissociation with the enzyme-free buffer compared to trypsin (Figures 2 and 3). Since natural tissue remodeling in vivo requires the breakdown of extracellular matrix by proteolytic enzymes [14-16], so as to enable cellular detachment, migration, and reattachment, it is plausible that the proteolysis of extracellular matrix and adhesion proteins may serve as a natural impetus or signal for cell reattachment to a new substratum. This is probably recapitulated in vitro through the proteolytic action of trypsin. With enzyme-free dissociation buffer, extracellular matrix and adhesion proteins synthesized by the cultured MSC are not digested away. Hence, there is a lack of natural impetus to reattach to a new substratum upon reseeding on new culture dishes. This could possibly explain the stark decrease in the proportion of viable reattached MSC upon dissociation with the enzyme-free buffer compared to trypsin (Figures 2 and 3).

The observation that freeze–thawing did not lead to an immediate significant decrease in cell viability (Figure 1), but resulted in a significant drop in the proportion of viable reattached MSC (Figure 2) is not surprising, considering the fact that post-cryopreservation apoptosis in MSC is well-documented [17]. Apoptosis takes a prolonged duration to manifest and would not be evident immediately after freeze–thawing.

In conclusion, our results show that the dissociation of MSC by enzyme-free buffer, as opposed to trypsin, causes a significant decrease in cell viability and reattachment of the dissociated MSC and, hence, is clearly unsuited for routine serial passage and propagation of MSC as used. Additionally, the data presented in this study would also have implications for the re-plating of dissociated MSC after cell-sorting, since enzyme-free cell dissociation is commonly used in fluorescence activated cell sorting (FACS) and magnetic affinity cell sorting (MACS ). Further studies are recommended to optimize the use of such buffers to dissociate MSCs for use in routine culture or for cell sorting purposes.
